# Tracheal injury diagnosed by a sudden increase in end-tidal carbon dioxide levels during mediastinoscopic subtotal esophagectomy: a case report

**DOI:** 10.1186/s40981-024-00695-3

**Published:** 2024-02-13

**Authors:** Natsuho Haraguchi, Yoshifumi Naito, Masayuki Shibasaki, Teiji Sawa

**Affiliations:** 1 Department of Anesthesiology, Fukuchiyama City Hospital, 231 Atsunaka-Cho, Fukuchiyama, Kyoto, 620-8505 Japan; 2 Department of Anesthesiology, Kyoto Prefectural University of Medicine, 465 Kajii-Cho, Kamigyo-Ku, Kyoto, 602-8566 Japan

**Keywords:** Mediastinoscopy, Esophagectomy, Pneumomediastinum, Tracheal injury, EtCO_2_, Asphyxiant gas, Hypoxia, Intoxication

## Abstract

**Background:**

Mediastinoscopic surgery for esophageal cancer facilitates early postoperative recovery. However, it can occasionally cause serious complications. Here, we present the case of a patient with a tracheal injury diagnosed by a sudden increase in end-tidal carbon dioxide (EtCO_2_) during mediastinoscopic subtotal esophagectomy.

**Case presentation:**

A 52-year-old man diagnosed with esophageal cancer was scheduled to undergo mediastinoscopic subtotal esophagectomy. During the mediastinoscopic procedure, the EtCO_2_ level suddenly increased above 200 mmHg, and the blood pressure dropped below 80 mmHg. We immediately asked the operator to stop insufflation and found a tracheal injury on the right side of the trachea near the carina by bronchoscopy. The endotracheal tube was replaced with a double-lumen tube, and the trachea was repaired via right thoracotomy. There were no further intraoperative complications. After surgery, the patient was extubated and admitted to the intensive care unit.

**Conclusions:**

Monitoring EtCO_2_ levels and close communication with the operator is important for safely managing sudden tracheal injury during mediastinoscopic esophagectomy.

## Background

In recent years, minimally invasive surgery has become increasingly common for the treatment of esophageal cancer. Mediastinoscopic esophageal surgery offers potential benefits for anesthetic management by avoiding differential lung ventilation or thoracotomy. Pneumomediastinum by carbon dioxide insufflation allows favorable expansion of the mediastinal space, and most esophagectomy surgeries are performed under a mediastinoscope at our institution [1]. Various complications, including recurrent laryngeal nerve paralysis, anastomotic leakage, and postoperative pneumonia, have been reported [2]. However, tracheal injury rarely occurs. Here, we report the case of a patient with tracheal injury diagnosed by a sudden increase in end-tidal carbon dioxide (EtCO_2_) during mediastinoscopic subtotal esophagectomy.

## Case presentation

A 52-year-old man (height, 160 cm; weight, 54 kg; body mass index, 21.0 kg/m^2^) having a diagnosis of esophageal cancer was scheduled for mediastinoscopic subtotal esophagectomy. He had undergone surgery for patent ductus arteriosus at 3 years of age. Preoperative examination revealed restrictive ventilatory impairment (%VC, 78%).

**Fig. 1:**
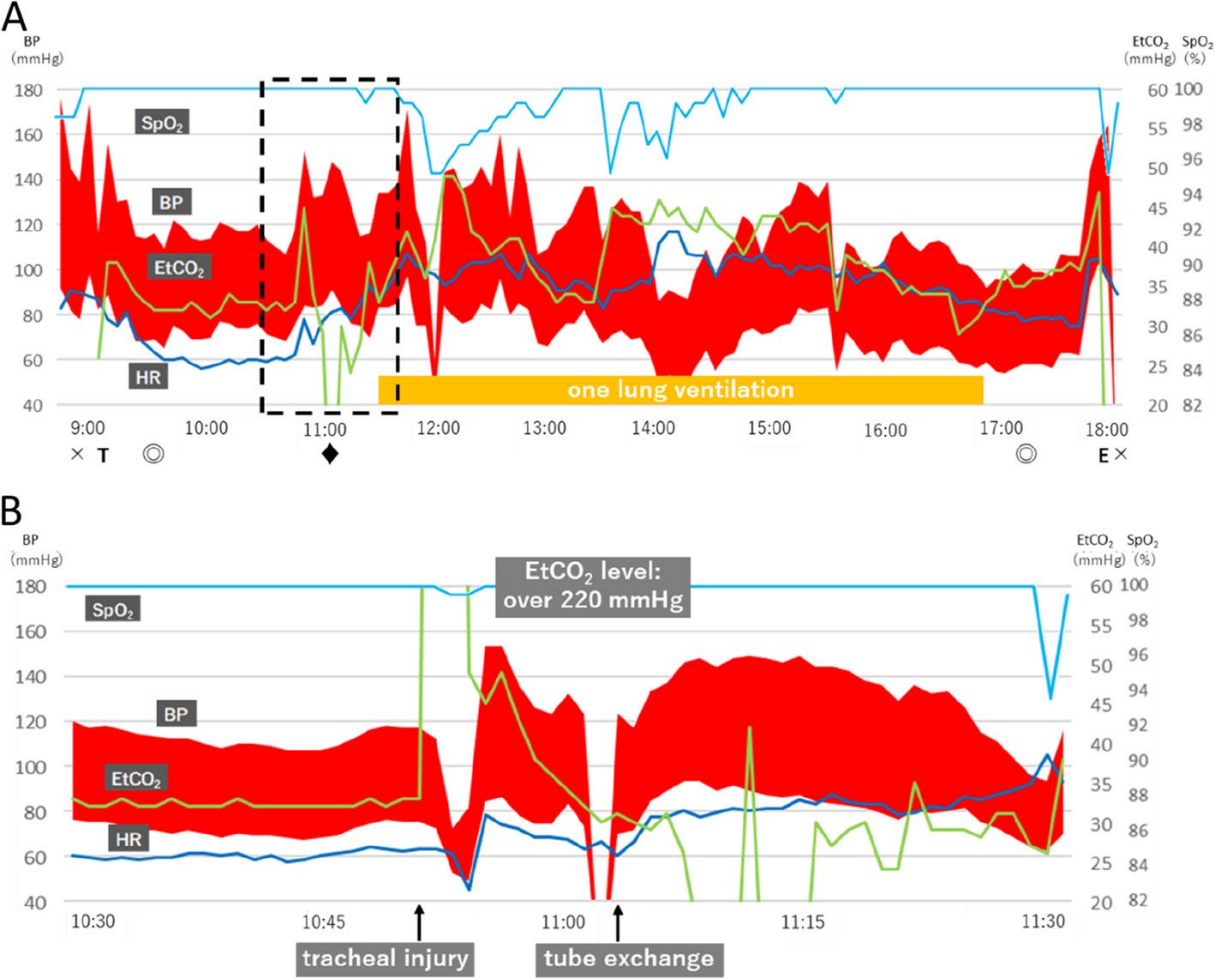
Intraoperative anesthesia record of the case. **A** An entire anesthesia record. A sudden increase in EtCO_2_ occurred at 10:51. × , anesthesia start/finish; T, intubation; ◎, operation start/finish; ♦, exchange of the endtracheal tube with a double-lumen tube; E, extubation; HR, heart rate; SpO_2_, percutaneous oxygen saturation; BP, blood pressure; EtCO_2_, end-tidal carbon dioxide partial pressure. **B** Anesthesia record from 10:30 to 11:30. The moment of tracheal injury, the blood pressure and the heart rate dropped to 72/52 mmHg and 45 beats/min. The percutaneous oxygen saturation remained at 99%

General anesthesia and epidural anesthesia were planned for the surgery (Fig. 1). The epidural catheter was inserted through the Th9–Th10 intervertebral space and placed 5 cm into the epidural space. General anesthesia was induced through rapid sequence induction with propofol 150 mg, fentanyl 100 µg, remifentanil 0.1 µg·kg^−1^·min^−1^, and suxamethonium 60 mg. Anesthesia was maintained with desflurane, fentanyl, and remifentanil. An electromyographic tracheal tube (NIM TriVantage™ EMG Endotracheal Tube; Medtronic Japan Co., Ltd., Tokyo, Japan) was used to avoid recurrent laryngeal nerve paralysis. Suxamethonium was used only for induction. Rocuronium was administered after the completion of the mediastinal procedure.

**Fig. 2:**
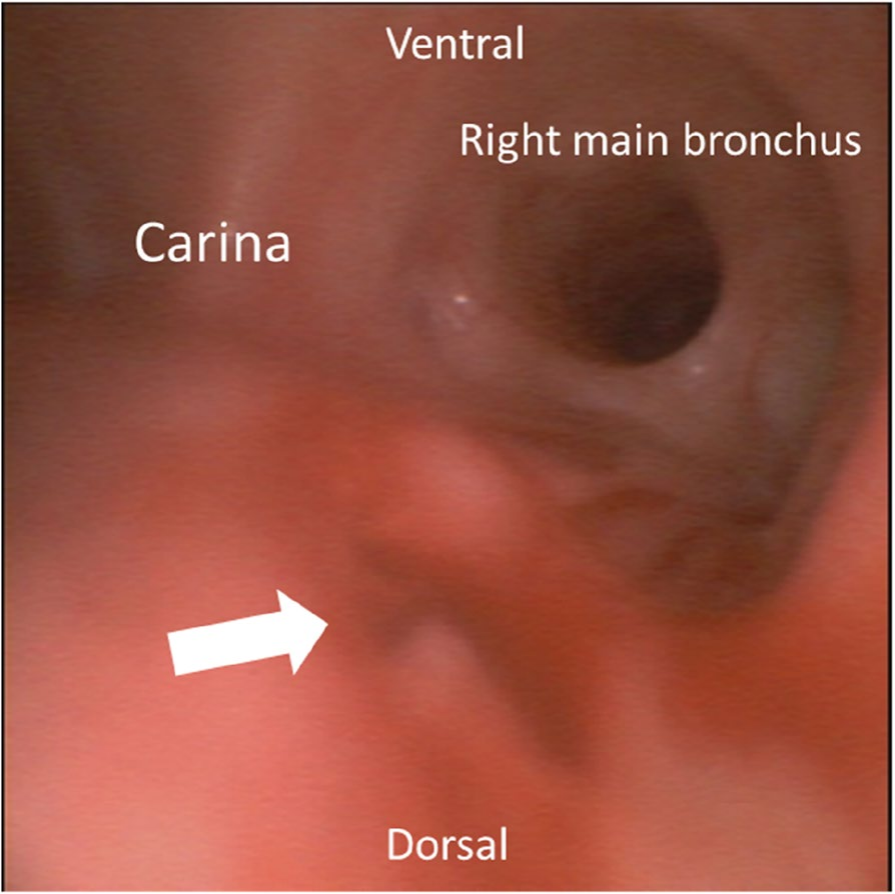
Bronchoscopic finding of the trachea during the surgery. The right side of the trachea near the carina was damaged (white arrow)

Approximately 1 h after the start of the surgery, the EtCO_2_ level suddenly exceeded its scale range (EtCO_2_, 227 mmHg; imCO_2_, 17 mmHg) when the operator was dissecting adhesions between the trachea and esophagus during the mediastinoscopy procedure. At the same time, the patient’s blood pressure and heart rate dropped to 72/52 mmHg and 45 beats/min, respectively. The percutaneous oxygen saturation (SpO_2_) remained at 99%. The operator was immediately notified that the EtCO_2_ level was abnormally high, and insufflation through mediastinoscopy was stopped. The patient’s vital signs improved with hyperventilation on 100% oxygen and administration of a vasopressor. The soda lime present in the anesthesia machine turned completely purple within minutes and had to be replaced. Suspecting a direct influx of carbon dioxide into the trachea caused by tracheal injury based on the sudden rise in EtCO_2_ during the tracheal manipulation, the surgery was interrupted and the trachea was observed by bronchoscopy. The right side of the trachea, near the carina, was damaged (Fig. 2). We administered 50 mg of rocuronium and replaced the NIM tube with a left double-lumen endobronchial tube to initiate left lung ventilation. The operator suspended the mediastinal procedure and initiated a laparoscopic transhiatal procedure. Afterward, the right thorax was opened, and the damaged area was filled with the greater omentum. As the repaired trachea showed no leakage and the patient’s vital signs remained stable, we proceeded to awaken the patient from anesthesia and extubate the double-lumen endobronchial tube. The patient was then transferred to the intensive care unit. The durations of surgery and anesthesia were 7 h 33 min and 9 h 12 min, respectively. Blood loss and urine output were 20 and 680 mL, respectively. The patient received 1500 mL of crystalloids and 1000 mL of colloids. Although anastomotic leakage was noted on postoperative day 11, the patient was treated with fasting and antibiotics (tazobactam/piperacillin) and was discharged from the hospital on postoperative day 54.

## Discussion

Mediastinoscopic subtotal esophagectomy offers several anesthetic advantages, particularly in patients with impaired pulmonary function. Avoiding differential lung ventilation helps minimize lung collapse associated with acute respiratory distress syndrome and severe oxidative stress [2, 3]. In addition, pulmonary function can be maintained by reducing postoperative pain [4]. This procedure is less likely to cause postoperative pneumonia and has been shown to improve the survival rate compared with thoracic and thoracoscopic surgeries [5]. However, the mediastinoscopic approach carries a high risk of complications, including severe blood loss due to injury to major blood vessels or the pericardial area, cerebral infarction resulting from compression of the brachiocephalic artery, pneumothorax, and tracheal injury [6, 7]. Recurrent laryngeal nerve paralysis is also a well-known complication. The NIM tube was used to prevent issues, and a bronchoscopic examination was conducted to confirm the presence of vocal cord paralysis after extubation. Hypercapnia or increased airway pressure may occur because mediastinoscopy in esophagectomy requires a pneumomediastinum to obtain a wide field of view for lymph node dissection [1].

In the present case, tracheal injury occurred during the pneumomediastinum procedure, resulting in increased EtCO_2_, decreased blood pressure, and decreased pulse rate. We believe that the changes in the patient’s vital signs were caused by reduced oxygen concentration due to the insufflation of a large amount of carbon dioxide from the injured site. The resulting hypoxia is commonly referred to as oxygen deficiency by asphyxiant gas. This occurs when elevated levels of the asphyxiant gas change the typical oxygen concentration. This phenomenon is usually noted in industrial areas, such as sewers, mines, and tunnels [8]. The safe limit of the oxygen concentration is 18%. At a concentration of 8–11%, there is a risk of fainting within a few minutes. If the concentration drops to 6–8%, fainting occurs rapidly, and if it drops below 6%, collapse occurs immediately [9]. A prior study conducted on rats reported that rapid asphyxia involving depletion of all oxygen resulted in respiratory arrest within 30–40 s, followed by cardiac arrest 2–3 min later [8].

Massive carbon dioxide insufflation causes hypoxia by asphyxiation and exerts a depressant effect on the respiratory and cardiac systems. Rapid exposure to high concentrations of CO_2_ results in the initial depression of blood pressure, a decrease in the heart rate due to direct depressant effects on the heart, and a decrease in peripheral vascular resistance due to direct vasodilation [10, 11]. Furthermore, carbon dioxide has an intoxicating effect and can be lethal, regardless of the oxygen concentration [11]. Carbon dioxide concentrations exceeding 10% can result in convulsions, coma, and death within minutes, whereas concentrations exceeding 30%, as in this case, can rapidly lead to loss of consciousness [12].

Regarding treatment, it is imperative to promptly avoid exposure to the asphyxiating gas and administer high concentrations of oxygen [13]. In this case, SpO_2_ did not decrease because the patient was immediately hyperventilated with 100% oxygen, although the end-tidal oxygen (EtO_2_) significantly reduced to 10%. If the situation had not been reported to the operator immediately and if carbon dioxide insufflation had continued, the oxygen concentration would have decreased further, potentially leading to cardiac arrest.

As the injury occurred on the right side of the trachea near the carina, the operation proceeded using left lung ventilation with a left double-lumen tube. Bronchoscopy should be used to promptly identify the site of tracheal injury and select the appropriate type of endotracheal tube to prevent air leakage from the injured site during positive pressure ventilation.

The incidence of tracheal injury during transhiatal esophagectomy has been reported to range from 0.4 to 1.4% [14, 15]. If a patient has a history of mediastinal surgery, there may be an increased risk of tracheal injury during dissection between the trachea and esophagus because of the possibility of tissue adhesions. It is unclear whether the patient’s previous surgical history of patent ductus arteriosus contributed to the injury.

Tracheal injury during mediastinoscopy is a rare and critical situation that requires immediate attention. To prevent serious complications, such as cardiac arrest caused by CO_2_ gas asphyxiation and intoxication, it is crucial to recognize the possibility of tracheal injury and to monitor the patient’s condition, particularly the EtCO_2_ levels, throughout the pneumomediastinum procedure. In addition, immediate communication with the operator is necessary if any unforeseen problems arise.

## Data Availability

Not applicable.

## Abbreviations

EtCO_2_: End-tidal carbon dioxide
CO_2_: Carbon dioxide
VC: Vital capacity
Th: Thoracic vertebra
NIM: Nerve integrity monitoring
EMG: Electromyogram
imCO_2_: Inspired minimum CO_2_
SpO_2_: Percutaneous oxygen saturation
ARDS: Acute respiratory distress syndrome
EtO_2_: End-tidal oxygen
